# Prevention of β Thalassemia in Northern Israel - a Cost-Benefit Analysis

**DOI:** 10.4084/MJHID.2014.012

**Published:** 2014-02-17

**Authors:** Ariel Koren, Lora Profeta, Luci Zalman, Haya Palmor, Carina Levin, Ronit Bril Zamir, Stavit Shalev, Orna Blondheim

**Affiliations:** 1 Pediatric Dept B and Pediatric Hematology Unit, Emek Medical Center, Afula, Israel.; 2 The Ruth and Baruch Rappaport School of Medicine, Technion, Israel Institute of Technology, Haifa, Israel.; 3 Emek Medical Centre, Afula, Israel, Affiliated with the Technion School of Medicine, Haifa, Israel.; 4 Hematology Laboratory, Emek Medical Centre, Afula, Israel.; 5 Unit of Genetics, Emek Medical Centre, Afula, Israel.

## Abstract

**Background:**

β Thalassemia major is characterized by hemolytic anemia, ineffective erythropoiesis and hemosiderosis. About 4% of the world population carries a Thalassemia gene. Management includes blood transfusions and iron chelation. However, this treatment is costly, and population screening may be significantly more cost beneficial.

**Purpose:**

The purpose of the current study is to analyze the cost of running a prevention program for β Thalassemia in Israel and to compare it to the actual expenses incurred by treating Thalassemia patients.

**Methods:**

Three cost parameters were analyzed and compared: the prevention program, routine treatment of patients and treatment of complications. An estimation of the expenses needed to treat patients who present with complications was calculated based on our ongoing experience in treating deteriorating patients.

**Results and Conclusions:**

The cost of preventing one affected newborn was $63,660 compared to $1,971,380 for treatment of a patient during 50 years (mean annual cost: $39,427). Thus, the prevention of 45 affected newborns over a ten year period represents a net saving of $88.5 million to the health budget. Even after deducting the cost of the prevention program ($413.795/year), the program still represents a benefit of $76 million over ten years. Each prevented case could pay the screening and prevention program for 4.6 years.

## Introduction

β Thalassemia major is a hemoglobinopathy characterized by chronic hemolytic anemia, ineffective erythropoiesis and progressive hemosiderosis. Thalassemia is considered the most frequent genetic disease in the world, about 4% of the world population carries a Thalassemia gene.^1^ In northern Israel the mean carrier frequency for β Thalassemia is about 2.4%; however, in villages with a primarily Arab population the frequency rises to 9%.^2^ Treatment of β Thalassemia major includes regular blood transfusions and iron chelation, to preclude early death.^3^,^4^ Disease management, which includes regular follow-up visits, is costly and carries a significant financial burden to health services.^5^

Approaches to Thalassemia prevention include general population screening at school age, as reported in Thailand;^6^ premarital screening, as described in Middle East countries such as North Cyprus, where abortion is not generally accepted;^7^,^8^ and the screening of couples at marriage or in early pregnancy, as performed in Sardinia.^9^ In Israel β Thalassemia prevention is based on the screening of young couples at early pregnancy for carrier detection and subsequent prenatal diagnosis. This approach may be significantly less expensive and more cost effective than others.^10^ In recent years more couples in Israel attend community health clinics in request of premarital genetic counseling.

Fifteen years ago the cost-benefit of a national Thalassemia prevention program in Israel was calculated.^11^ The cost of caring for Thalassemia patients with a life expectancy of 25–35 years was assessed, and the cost of a prevention program estimated; the real cost of the latter was not known then, as such a program had not yet been implemented. Changes in the approach to treatment have since significantly increased the life expectancy of individuals with Thalassemia, by 50 years and even more.

The purpose of the current study is to analyze the cost of prevention of β Thalassemia based on data from a prevention program that has been carried out in northern Israel since 1987,^2^ and to compare this cost to the actual expenses incurred by treating patients in the Pediatric Hematology Unit at the Emek Medical Centre, Israel.

## Materials and Methods

### Prevention Program

**Figure 1:**
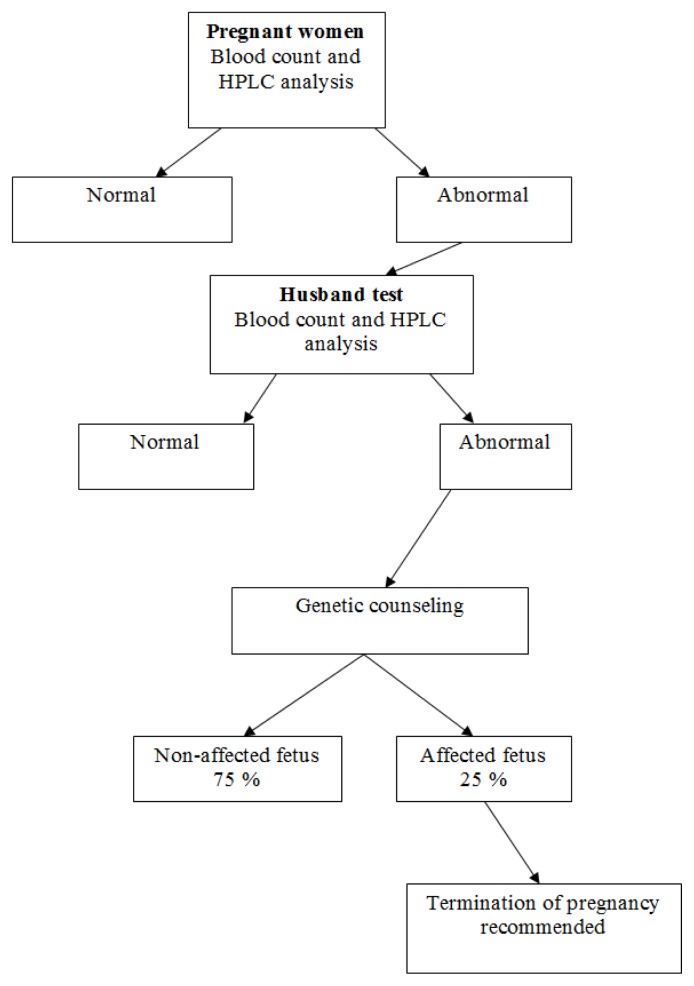
Prevention Program for β Thalassemia – Algorithm.

Since 1987, a screening program for the prevention of β Thalassemia and other hemoglobinopathies has been carried out in northern Israel. Pregnant women are screened at their first visit to community Mother and Child Health Clinics. Subsequently, husbands of affected women are screened, and couples identified as being at risk of having an affected offspring are referred for genetic counseling and prenatal testing (Figure 1).^2^ In the screening program all blood samples are analyzed by HPLC (High Performance Liquid Chromatography, cation exchange, Variant Hgb Testing, Biorad Co, USA), irrespective of the red blood cell indexes, in order to detect carriers with a MCV >78μ^3^ and carriers of Hgb S.^11^

Since the inception of the screening program, 71 β Thalassemia major and 25 β Thalassemia intermedia patients were diagnosed and treated at the Pediatric Hematology Unit in the Emek Medical Centre. Twenty-six β Thalassemia major patients succumbed to disease complications, most frequently iron overload, and died in the second or third decade of life.

### Cost Estimations

Three cost parameters were analyzed and compared:

- The prevention program.
- Routine treatment of patients with β Thalassemia major.
- Treatment of complications of β Thalassemia major.

- The annual cost of the prevention program was measured and calculated during the year 2011, including laboratory expenses with the HPLC method, expenses for laboratory personnel and expenses for a project manager (Table 1). We included the cost of the initial blood count, even though it is part of the routine tests of the first visit of pregnant women to Mother and Child Health Clinics.
- The cost for routine treatment of patients with β Thalassemia major was computed according to the actual management applied in the Pediatric Hematology Unit, based on the rates calculated and charged by Clalit Health Services, including the price of iron chelator Desferrioxamine (Desferal®, Novartis, provided by Teva, Home treatment Pharmaceuticals, Israel) or Deferasirox (Exjade®, Novartis, Israel) or Deferiprone (L1® - Ferrirprox® - ApoPharma – Canada provided by Luxemburg, Israel). In addition to the cost of treating β Thalassemia major patients, we included the cost of diagnosing new patients, including blood analysis of their parents (Blood count and HPLC analysis), blood transfusions [usually every three weeks], iron chelation (Desferal® or Exjade® or Deferiprone®) from age 2 years and the cost of annual follow-ups (Tables 2–4). The proportion of patients treated by Desferrioxamine, Deferiprone, combination therapy or Deferasirox was calculated according to the proportion of patients treated by each chelator during the year 2011.
- An estimation of the expenses of treating the complications of β Thalassemia was calculated based on our ongoing experience in treating deteriorating β Thalassemia patients in the Pediatric Hematology Unit. It is difficult to estimate the proportion of deteriorating patients since compliance with chelation treatment correlates inversely with the incidence of complications.

In our analysis, we did not include expenses for treatment of deteriorating patients due to poor compliance with chelation therapy, heart failure, endocrine workup and replacement therapy, diagnosis and treatment of osteoporosis and treatment of blood-related acquired infections. These expenses are presented in Table 5, with the proportion of patients presenting with each complication, based on our experience and compared with the rates reported in the literature.^12^ Also, expenses incurred directly by the families and direct compensation payments to the patients from the National Insurance Fund were not included in our analysis. Indirect costs related to poor quality of life and lost productivity are also not included in this analysis because of the wide variations between patients in these parameters.

The estimated costs are based on the fares calculated by the Health Insurance Services in Israel and include laboratory test and imaging costs, laboratory personnel, administrative staff and overhead for the general expenses that cannot be specifically calculated.

For the purpose of this study an exchange rate of 3.82 New Israel Shekel (NIS) for 1 US dollar was used, based on the average NIS/Dollar conversion rate for the year 2011.

## Results

**Figure 2:**
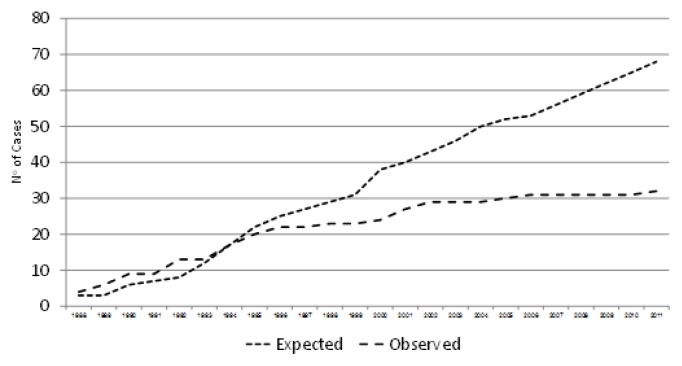
Observed and expected new cases of β Thalassemia in northern Israel – Years 1988 to 2011.

During a 24 year period (1987–2011), about 75.000 women were screened and more than 500 couples at risk of having an affected offspring were detected, representing a prevalence of 6.25 per 1000. Of 600 prenatal tentative diagnoses, about 110 affected fetuses were diagnosed and therapeutically aborted. During the screening period, 32 new β Thalassemia patients were diagnosed. All except one were born to women who were detected as carriers by the screening program but refused genetic counseling or prenatal diagnosis after counseling, or preferred not to perform a therapeutic abortion despite the diagnosis of an affected fetus (Figure 2). Of those fetuses, 22 were born in the first ten years that the program was implemented, while only four were born in the latter ten years.

As part of the screening program, about 5000 blood tests were performed annually. In the last ten years a mean of 26 couples/year were identified as being at risk of having an affected offspring, and prenatal diagnosis was subsequently performed. Assuming a 25% incidence of affected fetuses i.e. 6.5 affected fetuses/year, an average of four or five therapeutic abortions would be performed each year. The overall annual cost of running the prevention program was $413,795 (Table 1). The calculated cost for each affected fetus diagnosed was $63,660.

The cost of the diagnostic workup of a new β Thalassemia patient was $500 (Table 3). The routine follow-up for each patient was $1,410 annually. The yearly expenditure for treatment during the first two years of life without chelation therapy was $11,490. The introduction of chelation therapy from the third year increased annual expenses to $40,581. The overall mean cost per treatment-year was $39,427 including 2 T2*MRI heart and liver tests. The cost of performing splenectomy was about $4,116, but since only a few patients underwent splenectomy in recent years, the mean cost for this procedure in the whole cohort was negligible (Table 2). The total expenditure per patient for 50 years of life was $1,971,380, or an average of $39,427 per year (Tables 2 and 4). The costs of treating a patient with cardiac, endocrine or other complications were not included in the cost-effect calculations but are presented in Table 5. The rates of those complications are based on the experience in our Unit. Table 6 presents a method for calculation of treatment costs for a single β Thalassemia patient in different countries.

## Discussion

The burden of treating β Thalassemia major patients is substantial and the prevention program implemented in our region is of low cost compared to screening programs that employ molecular analysis for the detection of carriers.

Our assumption of life expectancy for β Thalassemia major is less than the 60 years estimated by Karnon et al.^5^ Modell et al.^13^ failed to show improvement in life expectancy in patients born with Thalassemia in the United Kingdom after 1965, or in those born after 1975. The authors estimated that not more than 50% of patients survive more than 35 years.^13^ However, 68% of β Thalassemia patients in Italy were alive at age 35 years.^12^ This may serve as evidence of the longevity that can be expected in developed countries that provide quality treatment and where compliance with chelation treatments is high. Our calculations are based on 100% compliance, which is an optimistic assumption since reported compliance rates range from 64 to 90%.^14^

Our calculated costs did not include treatment of deteriorating patients due to poor chelation compliance, such as cardiac or endocrine treatment or treatment of osteoporosis and blood related acquired infections. The expenses for such treatments can be easily calculated for a single patient, but the proportion of patients that present with such complications may differ considerably from country to country, depending on the quality of available medical treatment and compliance with chelation treatment. Other expenses not included in our cost analysis are the expenses paid directly by the families of the patients, such as travel to the hospital, loss of work days and compensation by the National Welfare Fund. Indirect costs related to the poor quality of life of such individuals were also not included in this analysis.

Healthcare expenses for a new Thalassemia patient in our study were 4 fold higher than calculated in Israel by Ginsberg et al 15 years ago.^15^ They calculated a total expenditure of $283,154 for life expectancy of 25–35 years, compared to our calculation of $1,971,380 for life expectancy of 50 years (ie: $9,438 and $39,427 per year, respectively). Since that study did not include a detailed calculation for treating a β Thalassemia patient, we cannot determine the source of the discrepancy between our calculations and theirs. Increased expenses following introduction of the new oral iron chelator, Deferasirox, significant improvements in quality of life and almost double life expectancy of patients with β Thalassemia are factors that may explain the differences.

Our calculations of treatment costs are higher than those estimated by Karnon et al for treating one β Thalassemia patient in the United Kingdom, $1,245,030, assuming a 60 year life expectancy (ie: $20,750 per year), and considerably higher than the annual cost of $9,168, as calculated by Cronin et al, also from the United Kingdom.^16^ Other recent studies from the United States calculated the annual cost of treating β Thalassemia patients with combination therapy of Desferrioxamine and Deferiprone at $22,199 in uncomplicated patients and $55,690 in patients with complications related to iron overload.^17^ When Deferasirox was used as the chelator treatment, the annual cost ranged from $24,400 to $53,095 in patients without complications^18^ and the total treatment costs for the life expectancy of 34.4 years was $1.8 million. This figure is similar to the one that we calculated ($1.97 million) for a 50 year life expectancy, and to that calculated in 1984 in Quebec, Canada.^19^ The purchase power in Israel and the United Kingdom are similar ($77.70 and $89.96, respectively), while higher in Canada and the United States ($112.10 and $140.80, respectively).

In Thailand, expenses to treat β Thalassemia patients are significantly lower than in the western world, probably due to less frequent blood transfusions and to the administration of low chelator doses. The costs reported were only $7,604 per year.^20^ The difference can be explained by a significantly lower purchase power, $35.56 compared to western countries.

The systematic screening of pregnant women at the Mother and Child Health Care Clinics in Israel precluded the need to establish a new system of clinics dedicated to screening. This reduced program costs. A similar screening program is currently implemented on a national basis, but the database for information analysis is not in operation, thus the data presented herein is based on the experience in northern Israel only.

Several means exist for instituting a prevention program of genetic disease. The least effective is the public distribution of information.^1^,^21^ Compulsory screening is the most effective method but not ethical in democratic countries. In countries where prenatal diagnosis is not permitted, compulsory screening and prevention of marriages between detected carriers is often utilized to reduce the incidence of genetic disease.^8^,^22^ Ginsberg et al^15^ estimated at $291,000 the cost of instituting an educational program for β Thalassemia prevention among the non-Jewish population in Israel, which is the population at highest risk in this country; the prevention of one affected fetus each year could cover this investment. However, educational programs alone cannot be cost beneficial and efficient enough to provide timely results to populations with a high incidence of carriers, especially when religious beliefs and traditions conflict with screening and intervention. In the program instituted in northern Israel, a significant decrease in the rate of affected newborns during the recent years, reflects the population’s collaboration with the program. In the last ten years (2001–2011), only four new patients with β Thalassemia major were born, only one of them in the last five years.

We showed that the cost of preventing one affected newborn is $63,660 compared to $1,971,380 for treatment of a β Thalassemia patient for 50 years (annual cost of $39,427). Thus, the prevention of 45 affected newborns over a ten year period represents a net saving of $88.5 million to the health budget. Even after deducting the costs of operating the prevention program in our region ($431.795/year, $20 million for 50 years) and the costs of treating the four newborn patients for 50 years ($8 million), the program still represents a substantial benefit of $60 million. Each prevented case could cover the costs of the screening and prevention program for 4.6 years. A study from Canada calculated the cost for each prevented case at $6700; however, that study was based on a small cohort of 6,748 persons in a high risk community.^19^ Our program provides universal screening; more than 80,000 pregnant women were screened since 1987. Increased costs over the last thirty years and the large experience of our medical center and healthcare system may explain the differences between the studies.

Our calculations are based on the social characteristics, gene frequencies and birth rate of the high risk population in northern Israel. Similar carrier rates were found in other non-Jewish populations, in the country. Specifically, tailored screening programs,^6^–^9^,^23^ including educational and voluntary pre-marital or pre-conception screening for populations of high socioeconomic levels, can reduce the cost of universal screening. We have shown the cost benefit of a national program for the screening of pregnant women for prevention of β Thalassemia in Israel, which may be applicable to countries with similar social settings. An additional benefit of such programs is the provision of prenatal counseling for future pregnancies without the need for further screening.

The total expense for treatment of deteriorating β Thalassemia patients is difficult to estimate. Incidence rates of complications have decreased significantly due to improvement in compliance with chelation treatment and to the high quality of blood units transfused. Nevertheless, the rates for such complications in our population are similar to those reported in 2004 from Italy.^12^ Because of the difficulty in computing the expenses of complications, we did not take them into account. Stem cell transplant is capable of curing thalassemia, but it is limited to eligible patients. The reported cost for each transplant varies from $100.000 to 200.000 in the United States and in Thailand^24^–^26^ to about $50.000, as is estimated in Israel, though no detailed data were calculated in Israel. Considering that the cost of one transplant is equivalent to one year of treatment, a successful transplant can certainly be cost effective.^24^ Since only a small proportion of thalassemia patients have been transplanted around the world, and calculation of the impact of stem cell transplant on the overall expenses of treating a cohort of patients is difficult, we did not include these calculations in our study.

The calculations presented herein also did not take into account the burden to the national economy in terms of welfare payments; loss of work capacity and educational support needed. According to one estimation, published 15 years ago, less than 10% of Thalassemia patients in Israel are employed.^15^ Adding the expenses of treatment for deteriorating patients and welfare payments would further raise the cost-effectiveness of the prevention program.

Two factors, the introduction of the oral chelator, Deferasirox, (Exjade ®, Novartis, Switzerland) and the improvement in survival beyond age 50, while raising the costs for treating Thalassemia patients, increase the attractiveness of the preventive program.

## Conclusions

This study showed that each new β Thalassemia patient born incurred an excessive budget of about $2 million for a life expectancy of 50 years. Such a budget could fund a prevention program for 4.6 years and prevent at least 31 affected patients.

Based on our calculations, and according the nationwide Mother and Child Health Care Clinics in Israel, implementation of a national β Thalassemia prevention program appears to have a high benefit- cost ratio. Benefits to society include, in addition to the direct financial savings of millions of dollars, the saving of hundreds of blood units, work power, compensation fees, treatment of endocrinological and cardiac complications and treatment of intercurrent complications and expenses during the phase of terminal deterioration.

## Figures and Tables

**Table 1:** Calculation of the expenses required for running a prevention program for β Thalassemia per year, based on data from 2011.

Test	No. of tests/year	Cost/test ($US)*	Cost/year ($US)
**Carrier screening**			
Blood count	5000	9.95	49,750
HPLC	5000	42.15	210,750
**Husband testing**			
Blood count	93.5	9.95	930
HPLC	93.5	42.15	3,941
**Prenatal diagnosis**			
Genetic counseling	26 couples	762	19,812
Gynecological ultrasound	26	438	11,388
Chorionic villus sampling	24	639	15,336
Amniocentesis	2	2651	5,302
DNA analysis	26	1914	49,764
Therapeutic abortion	4.5	4044	18,198
**Coordinator**			28,624
**Total annual cost**			413,795
**Cost per-prevented case**	6.5 cases/year		63,660

HPLC: High Performance Liquid Chromatography.

* Based on the rates calculated and charged by Clalit Health Services.

**Table 2:** Calculation of the expenses for diagnosis and treatment of one β Thalassemia patient for life expectancy of 50 years.

Test	Cost ($US) *

Diagnosis workup	**500**

Annual treatment during the first 2 years (blood transfusions)	10,080
Annual routine examinations/year + social worker	1.410

**Total cost from diagnosis to age 2 years**	**22,980**

Annual treatment from age 3 to age 50 years (Blood transfusions, iron chelator treatment, etc)	39,082
Annual routine examinations/year + social worker	1,410
Two T2* MRI studies (**)	4,284

**Total cost from age 3 to age 50 years**	**1,947,900**

Splenectomy (Procedure cost: $4116, not included in total treatment cost, see text)	4,116

**Total cost for life expectancy of 50 years**	**1,971,380**

**Mean annual cost**	**39,427**

(*) Based on rates calculated and charged by Clalit Health Services.

(**) Including two T2^*^MRI heart and liver tests during a lifetime.

**Table 3:** Detailed costs for initial diagnosis of a β Thalassemia patient.

Procedure/Test	Times/year	Cost/year ($US)*
**Initial Diagnosis**		
Complete blood count	1	9.95
HPLC	1	42.15
**Parents’ diagnoses**		
Complete blood count	2	19.90
HPLC	2	84.30
**Initial workup**		
G6PD quantitative test	1	101.00
Coombs direct test	1	50.79
Serum iron	1	14.92
Serum transferrin	1	69.11
Serum ferritin	1	11.52
Coverage (24%)		96.94
**Total costs for initial diagnosis**	**1**	**500.87**

HPLC: High Performance Liquid Chromatography.

(*) Based on rates calculated and charged by Clalit Health Services.

**Table 4:** Detailed annual costs for the treatment of a single β Thalassemia patient.

Procedure/Test	Frequency	Cost/unit/($US)*	Cost/year ($US)
Physician examination and blood transfusion	Every 3 weeks/18 per year	354	8,128
Social worker	Monthly/12 per year	20	242
Iron chelation (Desferrioxamine + Deferiprone) (10% of the patients)	2 gr/day x 6 days/week Deferiprone 3000 mg day x 7 days/week	192.15 week	9,992
Deferasirox (90% of the patients)	1061 mg/day	2216/month	26,597
Vitamin C	100 mg/day		14.73
Folic acid	1 tab/day		4.78
**Regular follow up tests**			
Liver and renal function	Monthly (**)	23.5	283
Serum iron	Once a year	14.92	14.92
Serum transferrin	Once a year	69.11	69.11
Serum ferritin	4 times/year	11.5	46
Serum folate	Once a year	50.96	50.96
Serum vitamin B_12_	Once a year	55.76	55.76
TSH	Once a year	13.35	13.35
Hepatitis C virus	Once a year	17.80	17.80
Anti HBsAb	Once a year	35.08	35.08
HIV test	Once a year	6.54	6.54
Audiometry	Once a year	69.11	69.11
Ophthalmologic examination	Once a year	69.11	69.11
Echocardiogram	Once a year	162.3	162.3
Influenza vaccination	Once a year	3.14	3.14

(*) Based on rates calculated and charged by Clalit Health Services.

(**) Requested for patients treated by Deferasirox.

**Table 5:** Expenses for treating complications of β Thalassemia major.

Complication	Incidence *	Principal Treatment	Cost/year ($US)	Treatment Years **	Total Lifelong Costs (50 years)
Hypothyroidism	9.6 % (10%)	Thyroxin	409	27	11,053
Hypoparathyroidism	5 % - (N.D.)	Ca + αD_3_	569	27	15,369
Insulin-dependent diabetes mellitus	7.6 % (6.4%)	Insulin	3,707	16	59. 317
Cardiomyopathy with congestive heart failure and or arrhythmias	10 % (6.8%)	ACE inhibitors Diuretics β Blockers	157	4 ***	628
Hospitalizations	42 days/yr/patient		15,392	4 ***	61,570
Intensification of Chelation		Desferrioxamine 6 gr/day	18,.445	4 ***	73,822
Chronic hepatitis C treatment –1 year treatment		Peginterferon, Ribavirine	16,834	1	16,834
Osteoporosis, including bone density analysis	30 % (N.D.)	Biphosphonates Calcium Vitamin D	710	25	17,764

* Frequency based on Emek Pediatric Hematology Unit data.

( ): Frequency from Ref 7. N.D.: Not described in Ref 7.

** Estimation based on Emek Pediatric Hematology Unit data.

*** Maximal survival in a deteriorating patient or until complete recovery.

Hormonal replacement therapy is not included because of the wide diversity in the usage of drugs.

**Table 6:** A method of computation of annual costs for basic treatment of a single β Thalassemia patient. Treatment of complications is not included.

		A	B	C
Procedure/Test	Frequency	N^0^/yr	Cost/unit/($US)	Cost/year ($US)
Physician exam	Every 3 weeks	18	Local cost/unit	A x B
Blood transfusion	Every 3 weeks	18	Local cost/unit	A x B
Blood count	Every 3 weeks	18	Local cost/unit	A x B
Social worker	Monthly	12	Local cost/unit	A x B
Iron chelation (Desferrioxamine + Deferiprone – DFP)	2 gr/day x 6 days/week 3 gr/day/x7 days/week	312 365	Local cost/unit	A x B
Deferasirox	20 mg/kg/day	365	Local cost/unit	A x B
Vitamin C	100 mg/day	365	Local cost/unit	A x B
Folic acid	1 tab/day	365	Local cost/unit	A x B
Liver + renal function	Monthly	12	Local cost/unit	A x B
Serum iron + transferrin	Once a year	1	Local cost/unit	A x B
Serum ferritin	4 times/year	4	Local cost/unit	A x B
Serum folate	Once a year	1	Local cost/unit	A x B
Serum vitamin B_12_	Once a year	1	Local cost/unit	A x B
Thyroid hormone tests	Once a year	1	Local cost/unit	A x B
Hepatitis C virus	Once a year	1	Local cost/unit	A x B
Anti HBsAb	Once a year	1	Local cost/unit	A x B
HIV test	Once a year	1	Local cost/unit	A x B
Audiometry	Once a year	1	Local cost/unit	A x B
Ophthalmologic examination	Once a year	1	Local cost/unit	A x B
Echocardiography	Once a year	1	Local cost/unit	A x B
Influenza vaccination	Once a year	1	Local cost/unit	A x B
**Total annual cost**				**Sum of column C**
